# Effects of brand‐matched alcoholic and alcohol‐free and low‐alcohol drinks adverts on drink selections: A United Kingdom‐based randomised controlled trial in an experimental online supermarket

**DOI:** 10.1111/add.70210

**Published:** 2025-10-20

**Authors:** Ru Jia, Lauren Bandy, Emma Davies, Hannah Forde, Peter Scarborough, Rachel Pechey

**Affiliations:** ^1^ Nuffield Department of Primary Care Health Sciences, Medical Science Division University of Oxford Oxford UK; ^2^ School of Psychology, Social Work and Public Health Oxford Brookes University Oxford UK

**Keywords:** alcohol advertising, alcohol free, brand‐sharing advertising, low‐alcohol, NoLo, randomised controlled trial

## Abstract

**Background and aims:**

Restricting alcohol advertising may reduce alcohol consumption and related ill‐health. However, advertisements for alcohol‐free and low‐alcohol drinks (NoLos) with brand‐matched alcoholic versions are typically exempt from restrictions, which could lead to surrogate marketing (adverts for NoLo beverages also promoting brand‐matched alcoholic options). This study measured the impact of advertisements for brand‐matched NoLo beverages on product selections in a simulated online supermarket, in the UK.

**Design, setting, participants and intervention:**

We conducted a randomised controlled trial with 1638 UK regular alcohol consumers (aged 18–91), assigned to one of three groups: (1) alcohol adverts (*n* = 469), where participants viewed an advertisement (embedded within a video) for one of four alcoholic beverages; (2) NoLo adverts (*n* = 472), for one of four brand‐matched NoLo beverages; and (3) unrelated adverts (*n* = 697), for batteries. Participants then selected food and drinks for a barbecue in a simulated online supermarket.

**Measurements:**

We collected data on products selected in the simulated online supermarket (e.g. product name, category, quantity, energy). Risk of alcohol dependence was measured using the Alcohol Use Disorder Identification Test Consumption Questions (AUDIT‐C). The primary outcome was selection (yes/no) of advertised alcoholic and NoLo products. Secondary outcomes included the selection (yes/no) of any alcoholic or NoLo products. We also examined the recall of advertised brands and products through survey questions.

**Findings:**

Viewing NoLo, but not alcohol, advertisements statistically significantly increased the odds of selecting the advertised products, compared with unrelated advertisements [alcohol group: odds ratio (OR) = 1.62, 95% confidence interval (CI) = 1.03–2.53, Benjamini‐Hochberg‐adjusted *P* = 0.11; NoLo group: OR = 2.18, 95% CI = 1.24–3.91, Benjamini‐Hochberg‐adjusted *P* = 0.022]. Although the odds of selecting the alcoholic version of the advertised product were higher in those exposed to NoLo adverts vs. control (OR = 1.48, 95% CI = 0.94–2.33, Benjamini‐Hochberg‐adjusted *P* = 0.13), this association did not reach statistical significance. Fifty‐nine percent of participants in all three groups recalled the advertised brand. Among those who remembered the brand of advertisement, 96% in the alcohol group also correctly recalled the advertised product, while 44% in the NoLo group reported seeing an advert for the alcoholic version of the product (*X* ^2^ = 297.16, *P* < 0.001, df = 2).

**Conclusions:**

Exposure to advertisements for brand‐matched alcohol‐free and low‐alcohol drinks increases brand (over product) recall, but, while the direction of effects is consistent with these advertisements promoting the selection of alcoholic beverages, evidence of the impact on alcohol selection is inconclusive.

## INTRODUCTION

Alcohol is the third biggest risk factor for poor health globally [1, 2, 3], so interventions to reduce this burden are a priority.

Alcohol marketing is particularly problematic because the content of some adverts (e.g. comedy videos, prize draws, etc.) may be appealing to children [4]. Alcohol brands (e.g. Heineken) are also likely to engage in advertising on social media, reaching up to 91% of young people 15 to 24 years old [5], and recent evidence shows that online alcohol advertising may influence drinking behaviours among young people through their engagement with the brands [6, 7]. Elsewhere, evidence demonstrates that young people are likely to initiate drinking, and drink at a heavier level, following exposure to alcohol advertising [8, 9, 10].

Alcohol advertising has been restricted or banned in some countries [11, 12, 13]. A comprehensive alcohol advertising ban was estimated to be a highly cost‐effective alcohol‐related harm intervention [14]. Alcohol advertising regulations are still under development in most countries [15, 16] and are often opposed and undermined by the industry [17, 18]. Such regulations are usually based on the alcohol content of drinks rather than the drinks' brand.

In addition to the specific product being advertised (e.g. Heineken 0.0%), advertising may increase the appeal of a category (e.g. beer) or brand (e.g. Heineken) as a whole [19, 20, 21]. Brand advertising typically uses iconography of an established brand (e.g. name, imagery, slogan) to promote a new product or enter a new market category [22]. Advertising an alcohol‐free or low alcohol (NoLo) version of an alcohol product (surrogate marketing) within the same brand could trigger associations with the alcoholic variant to some extent [23]. Such effects may be explained by brand advertising affecting recognition and recall of a brand [24, 25]. When consumers perceive similarity, consistency or congruity between a parent brand (e.g. Heineken) and an extension product (e.g. Heineken 0.0%), they may experience increased positive attitudes and purchase intentions toward both the parent brand and extension products [26]. Existing research already demonstrates that brand advertising affects unhealthy food choices, particularly among children and young people, potentially contributing to overweight and obesity [19].

There is a lack of evidence on the impact of brand advertising of drinks on behaviours. Sales of NoLo products [defined in the United Kingdom (UK) as <1.2% alcohol‐by‐volume] achieved total value of £221 m in the United Kingdom in 2021, with trends suggesting this will rise [27]. NoLo advertising might, on the one hand, be a helpful strategy in promoting sales of these products. On the other hand, it might be used as a substitute for alcohol advertising. In response to restrictions on alcohol advertising, advertising for NoLo products using the same brand iconography as the ‘regular strength’ alcohol products owned by the same brand [e.g. 0.0% alcohol by volume (ABV) Heineken beer vs. regular Heineken beer] has been observed [28]. For example, during the 2022 European Rugby Champions Cup, branding for Heineken 0.0 was displayed during fixtures played in Ireland [29], although alcohol adverts were banned [30]. In these circumstances, the association between products sharing the same brand may mitigate the effectiveness of the restrictions on alcohol advertising if the advertising triggers brand associations. However, there is a need for more evidence on NoLo advertising and alcohol consumption.

In this study, we aimed to investigate the impact of brand‐matched NoLo advertising on purchasing behaviours, in a simulated online supermarket, in the United Kingdom. We had three sets of aims. The primary aim was to investigate the differences in the selection of the alcoholic and NoLo versions of the advertised product among people who were exposed to brand‐matched alcoholic adverts, and brand‐matched NoLo adverts, compared to unrelated adverts. Our secondary aims were to (1) investigate the impact of brand‐matched NoLo advertising through brand recall, a proxy measure of behaviour; and (2) investigate the differences in the selection of alcoholic and NoLo products regardless of brand among people who were exposed to different adverts. Our exploratory aims were (1) describe the public support for different policies involving alcohol and NoLo advertising; (2) investigate the differences in the total energy (Kcal) selected among people who were exposed to different adverts; and (3) explore whether the impact of NoLo adverts on selection of any alcoholic or NoLo products differs by risk of alcohol dependence.

## METHODS

This study was a three‐arm randomised controlled trial (RCT) in an online virtual supermarket where participants were asked to complete a shopping task, after having viewed an advertisement for either alcoholic beverages, NoLo beverages or a neutral product. This study is reported using Consolidated Standards of Reporting Trials (CONSORT) statement [31]. Ethics approval was granted 7 December 2023 by the Central University Research Ethics Committee, University of Oxford (Ref: R65010/RE016). The study protocol was pre‐registered on Open Science Framework (https://doi.org/10.17605/OSF.IO/2FVWC).

### Design

In this RCT, participants were randomised to watch a short video with an embedded advertisement under one of the three conditions:

1. Advertisement of one of four alcoholic products [alcohol group (AG)].
2. Advertisement of one of four brand‐matched NoLo products [NoLo group (NLG)].
3. Advertisement of a non‐food‐or‐drink related neutral product (unrelated advert group (UG)].

One of four brands was shown to participants in AG and NLG to account for potential random effects of brands. Randomisation was performed by the survey platform Qualtrics via computerised random number generation on a 2:2:3 basis with random block sizes. In the original protocol, we thought to conduct the randomisation on a 1:1:1 basis. However, in the sample size calculation following our pilot study, we increased the size for UG to account for multiple comparisons against this group. Participants were recruited from independent research panels and were directed by automatic randomisation in the survey platform, to ensure the investigators were blinded to each allocation. Participants were only aware of the trial arm that they were exposed to.

### Participants

Our sample size of 1640 was based on an *a‐priori* power calculation to provide 90% power for the primary analysis (f^2^ = 0.026), which is described in Appendix SB. Data for the power calculation came from a pilot study with 200 participants.

Eligible participants were adults (age ≥ 18 years) living in the United Kingdom. Other inclusion criteria were: ability to speak and read English, willingness and ability to give informed consent, having access to a computer and the Internet and willingness to purchase and consume alcohol (measured by two questions: ‘Have you had any alcoholic drinks in the past month?’ and ‘Have you purchased any alcoholic drinks (e.g. a pint of beer at a pub) in the past month?’). Participants who answered ‘no’ to any of the eligibility questions were excluded from participation.

Participants were recruited from 3 July 2024 to 18 August 2024 through Dynata, a volunteer panel. We aimed to recruit participants that were representative of the United Kingdom in terms of age, gender and educational attainment. Participants were paid according to Dynata's standard rates.

### Procedure

After providing informed consent, participants completed a baseline survey on Qualtrics about their demographic characteristics (e.g. age, sex, gender, ethnicity, education, household income, household size) and lifestyle factors (e.g. typical weekly cost of grocery shopping, frequency of shopping for groceries online, whether or not they were willing to purchase and consume alcohol) (Appendix SB), before they were randomised to study conditions.

After randomisation, participants in all trial arms were shown a non‐food‐or‐drink related neutral video. All participants were asked to watch this video. A 10‐second advertisement was embedded at the start of this video. In total, the advertisement and video were 48 seconds long. To ensure the chance of exposure to the whole video, participants were unable to continue the study unless they stayed on this page for at least 48 seconds.

Next, participants were redirected to a simulated online supermarket to complete a shopping task, with the explanation: ‘This is not a real online supermarket. You will not be asked to spend any of your own money and you will not take home any groceries’. In the shopping task, participants were asked to imagine they were shopping for a barbecue with family and friends. They were asked to select at least 10 products including food, drinks and snacks. No limits on the budget were given. The experimental online supermarket platform (www.woodssupermarket.co.uk), hosted by the University of Oxford, emulates a real online supermarket for research purposes relating to food purchasing interventions (for details, see Figure SA2 and Table SA1). The site is populated with approximately 8708 unique supermarket products that were available to purchase in May 2022, taken from foodDB, a database of food and drinks available for purchase in six United Kingdom online supermarkets [32]. Participants interact with the site in a similar manner to a real online supermarket, but do not spend money or receive their selected products. All advertised products, except the battery, were available in the online supermarket.

On completion of the shopping task, participants were redirected to Qualtrics to complete a short post‐intervention survey on whether they could recall the advertisement they were exposed to, the acceptability of advertising restrictions, their perceptions of the intervention in the online shopping task and alcohol use.

### Intervention

**FIGURE 1:**
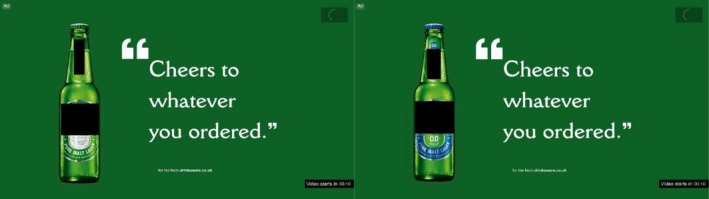
Examples of brand‐matched alcoholic (left) and alcohol‐free or low alcohol (NoLo) (right) drink adverts.

The adverts were created by the authors using existing product images and advertising slogans. Examples of the brand‐matched alcoholic and NoLo drink adverts, for AG and NLG respectively, are shown in Figure 1. For UG, participants saw an advertisement of a neutral product (i.e. batteries, see Figure SA1). Batteries were chosen for the neutral advert as they were non‐food‐or‐drink related, and therefore, unlikely to affect participants' food and drink choices in the shopping task. Advertisements were embedded in a video of how pencils are made (neutral video) to conceal the focus of the study from participants. The video had an audio component (i.e. upbeat music background without lyrics). The music started playing after the 10‐second advertisement, when the neutral video started, to mimic a naturalistic setting. All videos had the same music background. Details of how the video and adverts were presented to participants are described in later sections.

### Measures

#### Food and drink selection

Details of the food and drink products (e.g. product name, category, quantity, Kcal/100 g) present in baskets when participants reached ‘check‐out’ were recorded.

#### Recall of advertisement, brands and products

We measured recall of advertisements, brands and products as proxy measures of the effects of advertising on purchasing behaviours. Two questions were used, one for recall of advertisement, and one for recall of brand and product. Details of the questions, response options and interpretations of response are in Table SB1.

#### Alcohol use and risk of alcohol dependence

Alcohol use and risk of alcohol dependence was measured by the Alcohol Use Disorder Identification Test Consumption Questions (AUDIT‐C) [33].

#### Policy support

We included policy support measures to explore public opinions toward brand‐matched NoLo advertising, and policies aimed at restricting alcohol and NoLo advertising. Policy support was assessed on a 7‐point scale, adapted from Brown *et al*. [34], using the following two questions: ‘Do you support or oppose this policy?’ and ‘How acceptable do you find this policy?’. We, then, combined scores from the two questions to create an aggregate score of support. We assessed five policy scenarios: companies being able to advertise alcohol with no restrictions specified; companies being able to advertise brand‐matched alcohol‐free products; companies being able to advertise alcohol‐free products to young people and children <18 years old; restrictions on alcohol advertising without explicitly restricting brand advertising; and restrictions on alcohol advertising including alcohol‐free products that share the same branding as an alcoholic product.

### Analysis

Analyses were conducted in RStudio (version 2023.06.0 + 421). Outliers were defined as individuals who selected a single product for ≥50 times in the shopping basket, and were removed from all analyses (*n* = 2). A generalised linear model (GLM) was used to examine the differences in demographic characteristics and randomisation allocation between participants who completed the study and those who dropped out.

Primary outcomes were (1) selection of the alcoholic version of the advertised product; and (2) selection of the NoLo version of the advertised product. As the UG did not have a targeted brand, to allow comparison between UG and NLG/AG, we randomly allocated participants in UG to one of the four groups, each corresponding to a brand shown in NLG and AG, as the individual control group for each brand. The randomisation was conducted using a random assignment function in R (base). For the primary analysis, multinomial regression models compared the likelihood of selecting the alcoholic and NoLo version of the advertised products between the three study groups. A sensitivity analysis on the primary analysis was conducted using generalised linear mixed‐effect model (GLMM) (family = binomial, link=‘logit’) exploring the effect of intervention groups, with random effects assigned to the groups that saw adverts for the four different brands.

Recall (proportions of participants between groups) of advertisement (yes/no/unsure), brands (yes/no) and products (yes/no) were examined with χ^2^ tests.

For secondary analyses, logistic regression models compared the likelihood of selecting any alcoholic or NoLo product, regardless of brands, between the three study groups.

For exploratory analyses, we descriptively reported the self‐reported support for different policy scenarios. Linear regression models compared the energy (Kcal) from the alcoholic version of advertised products, the energy (Kcal) from the NoLo version of advertised products and total energy (Kcal) selected in shopping baskets, between the three study groups, given the energy in NoLo drinks may be lower.

We also conducted a logistic regression with an interaction term between cohort (NLG vs. UG) and risk of alcohol dependence categories, to explore the likelihood of selecting any alcoholic or NoLo product regardless of brands, in NLG compared with UG, by alcohol use and risk of dependence categories based on cut‐offs [33].

For pairwise comparisons, we also reported Benjamini‐Hochberg adjusted *P* values (BH‐adjusted *P*) in addition to unadjusted *P* values. Results should be interpreted primarily based on BH‐adjusted *P* values, with unadjusted values provided for transparency and context.

### Patient and Public Involvement

We conducted two patient and public involvement (PPI) focus groups during the planning of this study, and again after data collection, with 14 PPI public members of diverse demographic backgrounds who rated this study as relevant to population health. PPI members reviewed the simulated online supermarket and rated the tool as easy to use and reasonably naturalistic. However, they did acknowledge the limitation that participants did not have to spend money. The PPI panel also recommended inclusion of measures of risk of alcohol dependence, household income and educational attainments to ensure that the study was relevant to those who might be affected the most and to be inclusive.

## RESULTS

### Participant characteristics

**FIGURE 2:**
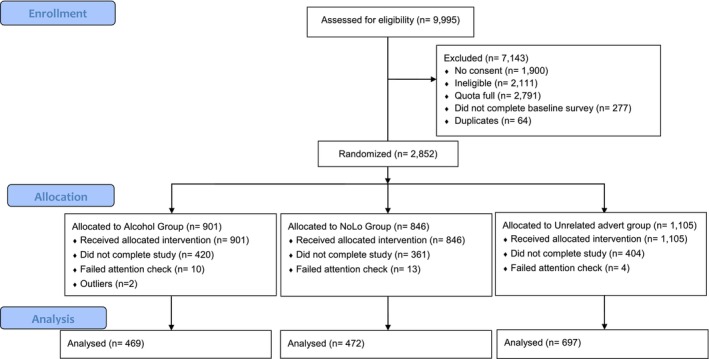
Consolidated Standards of Reporting Trials (CONSORT) flow diagram [31].

Of the 9995 individuals who completed the screening survey, 2852 met our eligibility criteria and were randomised. In total, 901 participants were allocated to AG, 846 participants were in NLG and 1105 were in UG. A total of 1640 participants completed the study (i.e. finished baseline survey, shopping task and follow‐up survey), with no missing data for primary and secondary outcomes. After removal of outliers (*n* = 2), primary analysis was conducted on data from 1638 participants (see CONSORT flow diagram, Figure 2).

Forty‐two percent (*n* = 1185) of participants who were randomised discontinued before finishing the study. Demographic characteristics of these participants are reported in Table 1. We conducted a GLM to examine whether demographic characteristics and randomisation allocation predicted participants dropping out. Participants in the intervention groups were more likely to discontinued the study compared to control (NLG vs. UG: OR = 1.34, 95% CI = 1.11–1.61, *P* = 0.002; AG vs. UG: OR = 1.55, 95% CI = 1.29–1.86, *P* < 0.001), but there was no differences in dropout in NLG and AG (NLG vs. AG: OR = 0.86, 95%CI = −0.34 to 0.04, *P =* 0.13). Dropout was more likely among those of older age (coefficient = 0.01, 95% CI = 0.001–0.012, *P =* 0.025) and with a higher household income (£43 K–66 K: OR = 0.76, 95% CI = −0.54 to −0.02, *P =* 0.038; ≥66 K: OR = 0.74, 95% CI = −0.58 to −0.03, *P =* 0.032, reference: £15–24 K).

**TABLE 1:** Characteristics of trial participants by study conditions.

Characteristics	Dropouts	Alcohol group	NoLo group	Unrelated advert group	Total	Missing
***n***	1185	469	472	697	1638	
Age, mean (SD)	52 (17)*	49 (17)	49 (16)	50 (17)	49 (17)	0
Gender, *n* (%)						
Women	663 (53)	240 (51)	255 (54)	345 (50)	840 (51)	0
Men	546 (46)	227(48)	216 (46)	349 (50)	791 (48)	0
Other/prefer not to say	6 (5)	3 (1)	<3 (0.2)	3 (0.4)	−(0.3)	0
Ethnicity, *n* (%)						
Asian or Asian British	36 (3)	21 (4)	32 (7)	26 (4)	79 (5)	0
Black, Black British, Caribbean or African	75 (6)	33 (7)	21 (4)	54 (8)	108 (7)	0
Mixed or multiple ethnic groups	68 (6)	28 (6)	19 (4)	37 (5)	84 (5)	0
Other/prefer not to say	11 (9)	7 (1)	<3 (0.4)	4 (0.6)	−(0.8)	0
White	995 (84)	380 (81)	398 (84)	576 (83)	1354 (83)	0
Education, *n* (%)						
No university degree	844 (71)	313 (67)	300 (64)	444 (64)	1057 (65)	0
University degree	335 (28)	155 (33)	170 (36)	248 (36)	573 (35)	0
Prefer not to say	6 (0.5)	<3 (0.2)	<3 (0.2)	5 (0.7)	−(0.5)	0
Household income, *n* (%)						
<£15 K	112 (9)	25 (5)	32 (7)	49 (7)	106 (7)	0
£15 K–£24 K	197 (17)	79 (17)	66 (14)	74 (11)	219 (13)	0
£24 K–£32 K	190 (16)	68 (15)	71 (15)	112 (16)	251 (15)	0
£32 K–£43 K	189 (16)	76 (16)	74 (16)	118 (17)	268 (16)	0
£43 K–£66 K	220 (19)*	101 (22)	103 (22)	143 (21)	347 (21)	0
≥£66 K	211 (18)*	92 (20)	102 (22)	168 (24)	362 (22)	0
Prefer not to say	66 (6)	28 (6)	24 (5)	33 (5)	85 (5)	0
Alcohol use disorders identification test						
Median (Q25, Q75)	NA	5 (3, 7)	5 (3, 7)	5 (3, 7)	5 (3, 7)	20

Abbreviation: NoLo, alcohol‐free or low alcohol.

*Statistically significant difference between participants who discontinued the study and participants who completed the study. Omitted numbers are to protect anonymity of participants.

Approximately half of study participants were females (51%, *n* = 840), and the mean age of participants was 49 years old, 83% of participants had a white background and with a median AUDIT‐C score of 5, indicating increasing risk of alcohol use disorders [33] (see Table 1). There were 20 missing values for AUDIT‐C scores (e.g. 5.4% of the sample), and missingness was determined to be consistent with missing completely at random (MCAR) via Little's test (*P* = 0.42), therefore, was addressed using listwise deletion in relevant analyses. There were no significant differences between intervention groups for demographic characteristics.

### Primary outcome: Selection of the advertised product

Estimates produced by our fitted logistic regression showed that participants in AG were more likely to select the alcoholic version of the advertised product, compared to UG (OR = 1.62, 95% CI = 1.03–2.53, *P* = 0.035) (see Table 2). However, these results did not remain significant after adjustment for multiple comparisons using the Benjamini–Hochberg method (BH‐adjusted *P* = 0.11).

**TABLE 2:** Study outcomes and characteristics of selected baskets by study conditions.

Outcomes	Alcohol group	NoLo group	Unrelated advert group	Total
Basket characteristics, mean (SD)				
Total no. of products in basket	20.3 (14.2)	20.1 (14.9)	19.6 (13.6)	19.9 (14.2)
Total price of products in basket (£)	59.2 (45.1)	57.5 (37.9)	56.0 (47.8)	57.3 (44.3)
Outcome (primary): selection of the alcoholic and NoLo version of the advertised product, *n* (%)				
Selection of alcoholic version of the advertised product	43 (9)	40 (9)	41 (6)	124 (8)
Selection of NoLo version of the advertised product	20 (4)	30 (6)	21 (3)	71 (4)
Selected both versions of the advertised product	9 (2)	6 (1)	5 (1)	20 (1)
Outcome (secondary): selection of the alcoholic and NoLo products regardless of brands, *n* (%)				
Selection of any alcoholic product regardless of brands	357 (76)	348 (74)	503 (72)	1208 (74)
Selection of any NoLo product regardless of brands	107 (23)	125 (27)	142 (20)	374 (23)
Selected both alcoholic and NoLo products regardless of brands	91 (19)	101 (21)	118 (17)	310 (19)
Outcome (exploratory): energy (Kcal) selected in basket, mean (SD)				
Energy (Kcal) selected in basket	12 297 (8884)	12 410 (8913)	11 791 (7961)	12 114 (8512)
Outcome (exploratory): policy support, mean (SD)				
Policy support^a^: alcohol advertising allowed	10.0 (2.5)	9.9 (2.5)	10.3 (2.4)	10.1 (2.4)
Policy support^a^: brand advertising allowed	11.0 (2.7)	11.3 (2.4)	11.3 (2.6)	11.2 (2.5)
Policy support^a^: NoLo advertising to young	6.0 (3.5)	5.6 (3.4)	5.9 (3.5)	5.8 (3.5)
Policy support^a^: restrict alcohol advertising	8.3 (3.2)	8.1 (3.2)	8.1 (3.1)	8.1 (3.2)
Policy support^a^: restrict brand advertising	7.9 (3.2)	7.8 (3.2)	7.7 (3.1)	7.8 (3.2)

Abbreviation: NoLo, alcohol‐free or low alcohol.

^a^ Sums of responses to two questions (‘Do you support or oppose this policy?’ and ‘How acceptable do you find this policy?’), on 7‐pt scales, with higher scores representing higher support.

**FIGURE 3:**
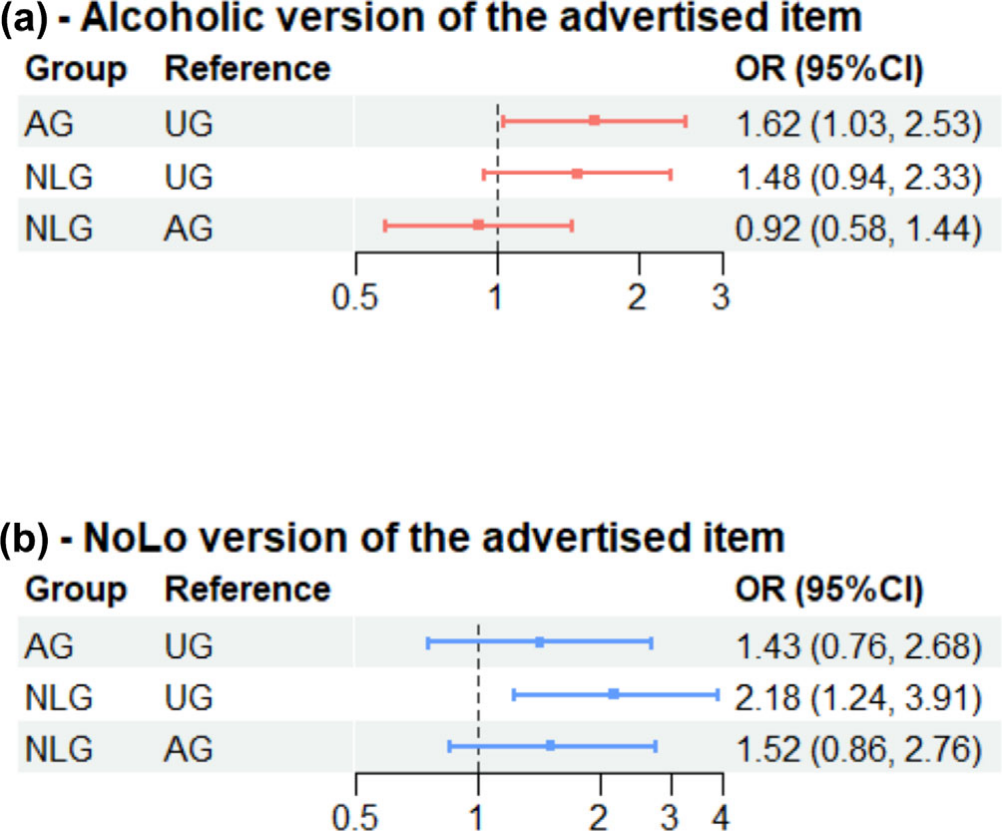
Selection of the alcoholic and alcohol‐free or low alcohol (NoLo) version of the advertised products by study conditions. AG, alcohol group; NLG, NoLo group; NoLo, alcohol‐free or low alcohol; UG, control.

For selection of NoLo version of the advertised product, participants in NLG were more likely to select the NoLo version, compared to UG (OR = 2.18, 95% CI = 1.24–3.91, BH‐adjusted *P* = 0.022 unadjusted *P* = 0.0072) (Figure 3).

### Primary outcome: Selection of alcoholic and NoLo versions having seen brand‐matched advertisements

There was no substantive evidence that NLG participants were also more likely to select the alcoholic version of the advertised product compared to UG (OR = 1.48, 95% CI = 0.94–2.33, BH‐adjusted *P* = 0.13, unadjusted *P* = 0.089) (Figure 3, Table SC1).

Similarly, there was no substantive evidence that AG participants were more likely to select a NoLo version of the drink than the UG (OR = 1.43, 95% CI =0.76–2.68, BH‐adjusted *P* = 0.26, unadjusted *P =* 0.26).

Results from the sensitivity analyses were broadly similar to the main results (see Table SC2).

### Secondary outcome: Recall of advertisement, brand and product

Approximately 73% of participants in AG and 71% of participants in NLG remembered seeing an advertisement during the study (see Table 3). Significantly fewer participants in UG (64%) recalled the advertisement (3 × 3 χ^2^ = 13.06, *P =* 0.012, d.f. = 4). Among those who recalled the advertisement, approximately 59% of participants in each trial group recalled the brand that was advertised to them. There were no significant differences between groups for recall of brand (3 × 2 χ^2^ = 0.096, *P =* 0.95, d.f. = 2). Among those who recalled the brand advertised, 96% of participants in AG recalled the product that was advertised to them (i.e. 4% incorrectly reported seeing the NoLo version), whereas just over half (56%) of participants in NLG recalled the product that was advertised to them (i.e. 44% incorrectly reported seeing the alcoholic version) (2 × 2 χ^2^ = 297.16, *P* < 0.001, d.f. = 2).

**TABLE 3:** Recall of advertisement, brand and product by study conditions.

Recall of advertisement	*n* (% of all participants in this cohort)		
	AG	NLG	UG
Yes	340 (73)	333 (71)	445 (64)
No	80 (19)	85 (18)	159 (23)
Unsure	40 (9)	54 (11)	93 (13)
Recall of brand			
Yes	278 (59)	280 (59)	408 (59)
No	191 (41)	192 (41)	289 (41)
Recall of the advertised product			
Yes	267 (57)	158 (34)	NA
No	202 (43)	314 (67)	NA

Abbreviations: AG, alcohol group; NA, not applicable; NLG, NoLo group; NoLo, alcohol‐free or low alcohol; UG, control.

We conducted an additional sensitivity analysis to explore the differences in the effect of NoLo adverts on the selection of NoLo version of the advertised product, between people who correctly recalled the advertised product and people who did not. Although underpowered, we found no conclusive evidence (z = 1.89, *P* = 0.06) that the effect size of NoLo advert on the selection of the NoLo version of the product was stronger among those who recalled the advertised product (OR = 1.90, 95% CI =0.87–4.20, *P* = 0.11), compared to those who did not recall the advertised product.

### Secondary outcome: Selection of any alcoholic and NoLo products regardless of brands

**FIGURE 4:**
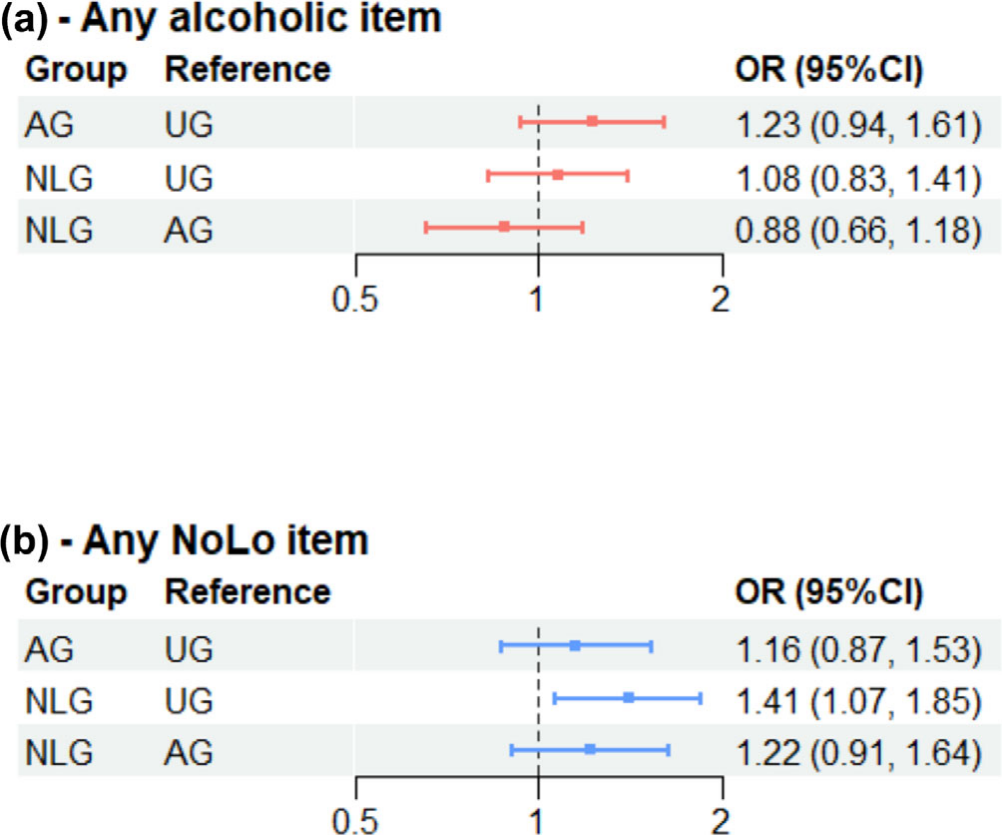
Selection of any alcoholic or NoLo products regardless of brands by study conditions. AG, alcohol group; NLG, NoLo group; NoLo, alcohol‐free or low alcohol; UG, control.

For secondary analyses, estimates produced by our fitted logistic regression suggested that there was no clear evidence that participants in AG were more likely to select any alcoholic products, regardless of brands, compared with UG (OR = 1.23, 95% CI = 0.94–1.61, BH‐adjusted *P* = 0.40, unadjusted *P* = 0.13) (Table 2). There is evidence that participants in NLG were more likely to select any NoLo products, compared with participants in UG (OR = 1.41, 95% CI = 1.07–1.85, BH‐adjusted *P* = 0.045, unadjusted *P =* 0.015) (Figure 4, Table SC3).

NoLo advertising had a very low and non‐significant impact on the selection of alcohol overall, and vice versa (participants in the NLG selecting alcohol, compared to UG: OR = 1.08, 95% CI = 0.83–1.41, BH‐adjusted *P* = 0.56, unadjusted *P =* 0.56; participants in the AG selecting NoLos, compared to UG: OR = 1.16, 95% CI = 0.87–1.53, BH‐adjusted *P* = 0.32, unadjusted *P =* 0.32).

### Exploratory analysis: Policy support

Of all policy scenarios, companies being able to advertise NoLo products to young people and children under the age of 18 received the least support (mean = 5.85, SD = 3.50) among our study population. The support for companies being able to advertise brand‐matched NoLo products was the highest (mean = 11.20, SD = 2.55) (Table 2). Exploratory (non–pre‐specified) paired‐sample *t* tests to exploring the differences in support between scenarios suggested significant differences in the support for each policy scenarios (see Table SC4).

### Exploratory analysis: Energy (Kcal) selected in basket

There was no evidence of differences between groups in the energy (Kcal) from the alcoholic version of advertised products, the energy (Kcal) from the NoLo version and total energy (Kcal) in shopping basket, between study groups (all *P >* 0.05), with the exception that NLG participants selected higher energy (Kcal) from NoLo version of advertised products than those in UG (coefficient = 0.20, 95% CI = 0.055–0.34, Table SC5).

### Exploratory analysis: Interaction between the impact of NoLo adverts and risk of alcohol dependence categories on the selection of any alcoholic and NoLo products regardless of brands

We conducted an exploratory (not pre‐specified) analysis of selections made by participants in the NLG, compared to UG, by risk of alcohol dependence, controlling for age, gender, ethnicity, education and household income. This explored whether NoLo advertising in particular may change behaviours toward alcoholic products among individuals at higher risk of alcohol dependence [35]. Full results are shown in Tables SC6 and SC7. We note that this is likely to be underpowered.

There was no evidence for a main effect on selection of any alcoholic products by study condition (OR for NLG compared to UG = 1.01, 95% CI = 0.66–1.54, *P* = 0.98). There was no substantive evidence for the main effect of risk of alcohol dependence: those who were at increasing risk (OR = 1.44, 95% CI = 0.96–2.18, BH‐adjusted *P* = 0.38, unadjusted *P* = 0.077), higher risk (OR = 1.44, 95% CI = 0.89–2.37, BH‐adjusted *P* = 0.54, unadjusted *P* = 0.14) of alcohol dependence and possible dependence (OR = 1.41, 95% CI = 0.59–3.77, BH‐adjusted *P* = 0.72, unadjusted *P* = 0.47) were more likely to select any alcoholic products, regardless of brands, compared with participants who were low‐risk.

There was no substantial evidence suggesting that individuals in NLG who were at increasing risk, higher risk of alcohol dependence and possible dependence were more likely to select any alcoholic product, or any NoLo product regardless of brands (all *P* > 0.05), compared to those at low risk and in UG (Table SC7, Figure SC1).

## DISCUSSION

We examined the impact of advertising the NoLo version of an existing alcoholic drink on the selection of both the alcoholic and NoLo versions of products, in a simulated online supermarket setting. We found strong evidence that NoLo beverage advertising might promote selection of NoLo products (BH‐adjusted *P* = 0.022), which, if used to replace alcoholic beverages, would benefit public health. The direction of effects was consistent with advertisements of NoLo beverages (that share the same brand of established regular‐strength alcoholic beverages) also promoting the selection of the alcoholic beverages themselves, albeit non‐significant (BH‐adjusted *P* = 0.13). However, we found that among those who correctly recalled the advertised brand, 96% of people in the alcohol group also correctly recalled the advertised product, whereas 44% of people in the NoLo group reported seeing an advert for the alcoholic version of the product (*P* < 0.001).

### Strengths and limitations

Our RCT had a relatively naturalistic setting including both subjective and objective measures of behaviours of a UK‐representative sample and drew on best practice guidelines for PPI [36]. Embedding adverts in a non‐food‐or‐drink‐related short video concealed the focus of the study. Furthermore, we ensured questions about the advertisement and advertising policy were only asked in the post‐intervention survey, at which point behavioural outcomes could not be changed.

Our study was conducted in a simulated online supermarket that closely resembled a real supermarket, but participants did not pay for nor receive products. Some participants' awareness of the experiment setting might have introduced response/demand bias. Both factors may influence the generalisability of our findings. The completion rate of the study was approximately 57%, lower than typically reported by a previous study with similar procedures (72%) [37]. The higher attrition rate in our study was likely because of redirection time from Qualtrics to the online supermarket and lost internet connection. Higher attrition rates among people with higher incomes, who are more likely to consume NoLo drinks [38], could result in fewer selections of NoLo products (both advertised and in general), undermining the potential effects of NoLo advertising. We included four of the biggest alcohol brands in our study (47). Although this might limit generalisability to other brands, our sensitivity analysis revealed that the main effects of advertising on selection of targeted drinks remain the same even after adjusting for the random effects of brands, suggesting that variation across brands may be small to minimal. Finally, we only explored a certain type of advert (i.e. visual image), but considering the different media (e.g. television, radio, social media) and types (e.g. visual, content) of advertising [8], future studies may extend this work and explore a wider range of advertising media and modalities.

### The effects of alcohol advertising

Previous evidence has established that alcohol advertising increases consumption. A meta‐analysis involving seven experimental studies of 758 participants found that viewing alcohol advertisements increased immediate alcohol consumption relative to viewing non‐alcohol advertisements by approximately 0.99 to 1.57 units [39]. However, all of these studies were conducted in student populations, albeit with a relatively wide age range (16–45 years). Another systematic review of 13 longitudinal studies of over 38 000 young people also concluded that alcohol advertising increases subsequent alcohol use [8]. Our findings correspond with existing literature that viewing alcohol advertisements may increase the odds of selecting alcoholic beverages in an experimental online supermarket, confirming the impact of alcohol advertising on both purchasing and consumption across the population.

### NoLo advertising: Potentials and concerns

NoLo beverages, as an alternative to alcoholic beverages, are proposed as a way to reduce population‐level alcohol consumption [40]. An analysis of household purchasing data from 64 280 British households between 2015 and 2018 found that the introduction of low‐ or no‐alcohol beer was associated with a sustained decrease in purchases of regular strength beer, indicating that NoLo beverages are purchased as substitutes, not in addition to, regular‐strength alcohol [41]. The role of advertising in shifting consumption from alcoholic products to no‐ and low‐alcohol alternatives remains uncertain. In our study, we demonstrated that advertising NoLo beverages has the potential to increase the odds of both selecting the advertised NoLo products and selecting NoLo beverages in general in a simulated online supermarket. This suggests that advertising NoLo beverages might effectively encourage the purchasing of NoLo beverages.

Nonetheless, a concern for advertising NoLo beverages is the effect of brand advertising on alcoholic beverage purchases [28]. In a previous study in Thailand where non‐alcoholic beverages (e.g. water and soft drinks) shared the same branding as an established alcoholic beverage (e.g. beer) were advertised, young people associated the advertisements primarily with the alcoholic beverage [21]. In our study, the NoLo beverage advertisements participants viewed closely resembled the brand‐matched alcoholic beverage advertisements. We observed that participants in the NoLo group were equally likely to recall the advertised brand as participants in the alcohol group, but a significant proportion of them did not recognise that the advert was for the NoLo version of the product. These results extended previous literature that in the context of advertising brand‐matched NoLo beverages, people's association with the advertising brand may be stronger than their association with the product itself [21]. As our inconclusive results suggest, its effects on behaviours are still unclear.

Among people at greater risk of alcohol dependence, we did not find conclusive evidence that viewing NoLo adverts altered selection of any alcoholic drinks, regardless of brands. However, the direction of effects was consistent with those at higher risk of alcohol dependence, or with possible dependence having increased selections, compared with those at low risk. Although these results are exploratory and likely to be underpowered, they point to the possibility that NoLo advertising may also encourage the purchasing of alcoholic beverages, particularly in those at risk of alcohol dependence. Confirmation and exploration in future studies is urgently needed, especially to determine whether NoLo advertising could have triggering effects for those vulnerable to alcohol dependence [42].

The World Health Organization has suggested restricting advertising associating no‐ and low‐alcohol beverages with full‐strength similar brand products [43]. Implementing this suggestion may have been slowed by an absence of evidence to demonstrate the impact of brand sharing advertising on purchasing and consumption behaviours. In this study, we found initial, albeit inconclusive, evidence that, in a UK population, brand sharing advertising may have the potential to influence behaviours in a manner that could favour the need for restricting brand sharing NoLo advertising. The potential effects of restricting such advertising as a population‐level intervention should be confirmed and explored further in future studies.

## CONCLUSION

This study found novel evidence that exposure to NoLo beverage advertisements may influence the recall, and potentially subsequent purchasing, of brand‐matched products. We also found that exposure to NoLo beverage advertisements significantly increased selection of NoLo beverages in general. These results first suggest the potential for interventions to encourage consumption of NoLos, if these act as substitutes to alcoholic beverages to reduce overall alcohol consumption. They also indicate the potential challenges to separate the effects of brand‐sharing advertising to reduce the population exposure to alcohol brands, which may also promote purchasing and consumption of alcohol.

## AUTHOR CONTRIBUTIONS

**Ru Jia:** Conceptualization (equal); data curation (lead); formal analysis (lead); investigation (equal); methodology (equal); project administration (lead); resources (equal); software (lead); validation (lead); visualization (lead); writing—original draft (lead); writing—review and editing (lead). **Lauren Bandy:** Conceptualization (equal); methodology (equal); visualization (equal); writing—original draft (supporting); writing—review and editing (equal). **Emma Davies:** Conceptualization (equal); methodology (equal); visualization (equal); writing—original draft (supporting); writing—review and editing (equal). **Hannah Forde:** Conceptualization (equal); methodology (equal); visualization (equal); writing—original draft (supporting); writing—review and editing (equal). **Peter Scarborough:** Conceptualization (equal); investigation (equal); methodology (equal); supervision (supporting); visualization (equal); writing—original draft (supporting); writing—review and editing (equal). **Rachel Pechey:** Conceptualization (equal); formal analysis (equal); funding acquisition (lead); investigation (equal); methodology (equal); project administration (supporting); resources (equal); supervision (lead); validation (equal); visualization (equal); writing—original draft (equal); writing—review and editing (equal).

## DECLARATION OF INTERESTS

We declare no conflicts of interest.

## Supporting information


**Figure SA1** Example of neutral adverts.
**Figure SA2** Example page from the experimental online supermarket.
**Table SA1** Number of products available by department and aisle in the experimental online supermarket.
**Table SC1** Logistic regression models for the selection of alcoholic and NoLo version of the advertised product by group.
**Table SC2** Generalised linear mixed‐effect models (GLMMs) for the selection of alcoholic and NoLo version of the advertised product by group with brands as random effect.
**Table SC3** Logistic regression models for the selection of any alcoholic and NoLo products regardless of brands by group.
**Table SC4** Paired‐sample t‐tests for the differences in support for each policy scenarios.
**Table SC5** Linear regression models for energy (kcal) selected in basket.
**Table SC6** Number (proportion) of participants in NLG by risk of alcohol dependence categories.
**Table SC7** Logistic regression models with an interaction term between cohort (NLG vs UG) and risk of alcohol dependence categories.
**Figure SC1** Selection of any alcoholic or NoLo products regardless of brands in NLG by risk of alcohol dependence measured by AUDIT‐C. NLG: NoLo group. UG: unrelated advert group.

## Data Availability

The dataset generated and analysed during the current study is available in the Open Science Framework repository.

## References

[1] Bellos S , Skapinakis P , Rai D , Zitko P , Araya R , Lewis G , et al. Cross‐cultural patterns of the association between varying levels of alcohol consumption and the common mental disorders of depression and anxiety: secondary analysis of the WHO collaborative study on psychological problems in general health care. Drug Alcohol Depend. 133(3):825–831. 10.1016/j.drugalcdep.2013.08.030 24156883

[2] Parry CD , Patra J , Rehm J . Alcohol consumption and non‐communicable diseases: epidemiology and policy implications. Addiction. 2011;106(10):1718–1724. 10.1111/j.1360-0443.2011.03605.x 21819471 PMC3174337

[3] Bryazka D , Reitsma MB , Griswold MG , Abate KH , Abbafati C , Abbasi‐Kangevari M , et al. Population‐level risks of alcohol consumption by amount, geography, age, sex, and year: a systematic analysis for the global burden of disease study 2020. Lancet. 2022;400(10347):185–235. 10.1016/S0140-6736(22)00847-9 35843246 PMC9289789

[4] Winpenny E , Patil S , Elliott M , van Dijk LV , Hinrichs S , Marteau T , et al. Assessment of young people's exposure to alcohol marketing in audiovisual and online media. Cambridge: Rand Europe; 2012. 158 p. Available from: https://citeseerx.ist.psu.edu/document?repid=rep1&type=pdf&doi=74163b10482fd055ab2d4899c927c1a04567d77f

[5] Winpenny EM , Marteau TM , Nolte E . Exposure of children and adolescents to alcohol marketing on social media websites. Alcohol Alcohol. 2014;49(2):154–159. 10.1093/alcalc/agt174 24293506 PMC3932831

[6] Atkinson AM , Ross‐Houle KM , Begley E , Sumnall H . An exploration of alcohol advertising on social networking sites: an analysis of content, interactions and young people's perspectives. Addict Res Theory. 2017;25(2):91–102. 10.1080/16066359.2016.1202241

[7] Niland P , McCreanor T , Lyons AC , Griffin C . Alcohol marketing on social media: young adults engage with alcohol marketing on facebook. Addict Res Theory. 2017;25(4):273–284. 10.1080/16066359.2016.1245293

[8] Anderson P , de Bruijn A , Angus K , Gordon R , Hastings G . Impact of alcohol advertising and media exposure on adolescent alcohol use: a systematic review of longitudinal studies. Alcohol Alcohol. 2009;44(3):229–243. 10.1093/alcalc/agn115 19144976

[9] Alhabash S , Mundel J , Deng T , McAlister A , Quilliam ET , Richards JI , et al. Social media alcohol advertising among underage minors: effects of models' age. Int J Advert. 2021;40(4):552–581. 10.1080/02650487.2020.1852807

[10] Carrotte ER , Dietze PM , Wright CJ , Lim MS . Who ‘likes’ alcohol? Young Australians' engagement with alcohol marketing via social media and related alcohol consumption patterns. Aust N Z J Public Health. 2016;40(5):474–479. 10.1111/1753-6405.12572 27624756

[11] WHO. World Health Organization . [cited 2023 Jul 26]. GHO|Global Health Observatory Data Repository (European Region)|Advertising restrictions on national television ‐ by country. https://apps.who.int/gho/data/node.main-euro.A1132?lang=en&showonly=GISAH

[12] Institute of Alcohol Studies . [cited 2023 Jul 26]. Alcohol and marketing. Available from: https://www.ias.org.uk/report/alcohol-and-marketing/

[13] World Health Organization . Global status report on alcohol and health 2018. Geneva: World Health Organization; 2018 [cited 2023 Jun 21]. 450 p. https://apps.who.int/iris/handle/10665/274603

[14] Anderson P , Chisholm D , Fuhr DC . Effectiveness and cost‐effectiveness of policies and programmes to reduce the harm caused by alcohol. Lancet. 2009;373(9682):2234–2246. 10.1016/S0140-6736(09)60744-3 19560605

[15] Berdzuli N , Ferreira‐Borges C , Gual A , Rehm J . Alcohol control policy in Europe: overview and exemplary countries. Int J Environ Res Public Health. 2020;17(21):8162. 10.3390/ijerph17218162 33158307 PMC7663832

[16] Esser MB , Jernigan DH . Policy approaches for regulating alcohol marketing in a global context: A public health perspective. Annu Rev Public Health. 2018;39:385–402. 29608872 10.1146/annurev-publhealth-040617-014711

[17] Maani Hessari N , Bertscher A , Critchlow N , Fitzgerald N , Knai C , Stead M , et al. Recruiting the “heavy‐using loyalists of tomorrow”: an analysis of the aims, effects and mechanisms of alcohol advertising, based on advertising industry evaluations. Int J Environ Res Public Health. 2019 Jan;16(21):4092. 10.3390/ijerph16214092 31652921 PMC6862254

[18] van Schalkwyk MCI , Maani N , Hawkins B , Petticrew M , Buse K . Reclaiming the narrative: Countering harmful commercial discourses. Health Promot Int. 2024;39(6):daae182. 39657150 10.1093/heapro/daae182 PMC11630776

[19] Boyland EJ , Halford JCG . Television advertising and branding. Effects on eating behaviour and food preferences in children. Appetite. 2013;62:236–241. 22421053 10.1016/j.appet.2012.01.032

[20] Smith R , Kelly B , Yeatman H , Boyland E . Food marketing influences children's attitudes, preferences and consumption: a systematic critical review. Nutrients. 2019;11(4):875. 10.3390/nu11040875 31003489 PMC6520952

[21] Kaewpramkusol R , Senior K , Nanthamongkolchai S , Chenhall R . Brand advertising and brand sharing of alcoholic and non‐alcoholic products, and the effects on young Thai people's attitudes towards alcohol use: a qualitative focus group study. Drug Alcohol Rev. 2019;38(3):284–293. 10.1111/dar.12910 30740803

[22] Bartram A , Harrison NJ , Norris CA , Christopher J , Bowden JA . Zero‐alcohol beverages and brand extensions: A vehicle for promoting parent alcohol brands? Aust N Z J Public Health. 2024;48(2): 100141. PMID: http://www.scopus.com/inward/record.url?scp=85188053222&partnerID=8YFLogxK 38503145 10.1016/j.anzjph.2024.100141

[23] Critchlow N , Holmes J , Fitzgerald N . Alibi marketing? Surrogate marketing? Brand sharing? What is the correct terminology to discuss marketing for alcohol‐free and low‐alcohol products which share branding with regular strength alcohol products? Addiction. 2025 Jan;120(1):4–6. https://onlinelibrary.wiley.com/doi/abs/10.1111/add.16504 38631701 10.1111/add.16504

[24] Khurram M , Qadeer F , Sheeraz M . The role of brand recall, brand recognition and price consciousness in understanding actual purchase Rochester, NY: Social Science Research Network; 2018 [cited 2025 Jan 9]. https://papers.ssrn.com/abstract=3215875

[25] Thoma V , Williams A . The devil you know: The effect of brand recognition and productratings on consumer choice. Judgm Decis Mak. 2013;8(1):34–44. 10.1017/S1930297500004484

[26] Deng Q , Messinger PR . Dimensions of brand‐extension fit. Int J Res Mark. 2022;39(3):764–787.

[27] Holmes J , Angus C , Kersbergen I , Pryce R , Stevely A , Wilson L . No‐ and low‐alcohol drinks in Great Britain: Monitoring report [Internet]. The University of Sheffield; 2024 Jan [cited 2025 Jan 16]. https://orda.shef.ac.uk/articles/report/No-_and_low-alcohol_drinks_in_Great_Britain_Monitoring_report/24893526/3

[28] Critchlow N , Moodie C , Houghton F . Brand sharing between alcoholic drinks and non‐alcoholic offerings: A challenge to Ireland's restrictions on alcohol advertising. Ir J Med Sci (1971 ‐) 2023 Aug;192(4):1975–1977. 10.1007/s11845-022-03161-0 36114361

[29] Highlights ‐ Leinster Rugby v Stade Toulousain ‐ Semi‐finals │Heineken Champions Cup Rugby 2021/22. 2022 [cited 2025 Jan 16]. Available from: https://www.youtube.com/watch?v=N7Ks2II1lT4

[30] Irish Status Book . Public health (alcohol) act 2018 [internet]. Dublin: Oireachtas; 2018 [cited 2025 Jan 16]. https://www.irishstatutebook.ie/eli/2018/act/24/enacted/en/html

[31] Schulz KF , Altman DG , Moher D , CONSORT Group . CONSORT 2010 statement: Updated guidelines for reporting parallel group randomised trials. BMJ. 2010;340:23. 10.3736/jcim20100702 20619135

[32] Harrington RA , Adhikari V , Rayner M , Scarborough P . Nutrient composition databases in the age of big data: foodDB, a comprehensive, real‐time database infrastructure. BMJ Open. 2019;9(6):e026652. 10.1136/bmjopen-2018-026652 PMC6609072 31253615

[33] Bush K , Kivlahan DR , McDonell MB , Fihn SD , Bradley KA . The AUDIT alcohol consumption questions (AUDIT‐C): an effective brief screening test for problem drinking. Ambulatory care quality improvement project (ACQUIP). Alcohol use disorders identification test. Arch Intern Med. 1998;158(16):1789–1795. 10.1001/archinte.158.16.1789 9738608

[34] Brown K , Hill KM , Smith J , Johansson M , Davies EL . Acceptability of alcohol‐free dance in place of traditional alcohol‐focused events. Health Educ J. 2021;80(3):300–312. 10.1177/0017896920973298

[35] Dumbili EW , Leonard P , Larkin J , Houghton F . Prioritising research on marketing and consumption of no and low (NoLo) alcoholic beverages in Ireland. Int J Drug Policy. 2025;139:104794. 40184695 10.1016/j.drugpo.2025.104794

[36] Price A , Schroter S , Snow R , Hicks M , Harmston R , Staniszewska S , et al. Frequency of reporting on patient and public involvement (PPI) in research studies published in a general medical journal: a descriptive study. BMJ Open. 2018;8(3):e020452. 10.1136/bmjopen-2017-020452 PMC5875637 29572398

[37] Jostock C , Luick M , Jebb SA , Pechey R . Changing the availability and positioning of more vs. less environmentally sustainable products: A randomised controlled trial in an online experimental supermarket. Appetite. 2024;200:107579. 38914261 10.1016/j.appet.2024.107579 PMC7618412

[38] Waehning N , Wells VK . Product, individual and environmental factors impacting the consumption of no and low alcoholic drinks: A systematic review and future research agenda. Food Qual Prefer. 2024;117:105163.

[39] Stautz K , Brown KG , King SE , Shemilt I , Marteau TM . Immediate effects of alcohol marketing communications and media portrayals on consumption and cognition: a systematic review and meta‐analysis of experimental studies. BMC Public Health. 2016;16(1):465. 10.1186/s12889-016-3116-8 27278656 PMC4899920

[40] Miller M , Pettigrew S , Wright CJC . Zero‐alcohol beverages: harm‐minimisation tool or gateway drink? Drug Alcohol Rev. 2022;41(3):546–549. 10.1111/dar.13359 34370881

[41] Jané Llopis E , O'Donnell A , Kaner E , Anderson P . Are lower‐strength beers gateways to higher‐strength beers? Time series analyses of household purchases from 64,280 British households, 2015–2018. Alcohol Alcohol. 2022;57(4):520–528. 35512687 10.1093/alcalc/agac025 PMC9270994

[42] Davies EL , Perman‐Howe P , Seddon J , Piatkowski T , Puljevic C , Barratt MJ , et al. Barriers to the use of no and low alcohol products in high‐risk drinkers. Drug Alcohol Rev. 2025 Mar;44(3):842–857. PMID: https://onlinelibrary.wiley.com/doi/abs/10.1111/dar.14006 39923234 10.1111/dar.14006 PMC11886542

[43] World Health Organization . A public health perspective on zero‐ and low‐alcohol beverages. Brief 10, 2023. World Health Organization; 2023. 22 p.

