# Supplementary material for: Effects of brand‐matched alcoholic and alcohol‐free and low‐alcohol drinks adverts on drink selections: A United Kingdom‐based randomised controlled trial in an experimental online supermarket
Source: Addiction. 2025 Oct 20;121(2):388–99. doi: 10.1111/add.70210 (PMC12779593; doi:10.1111/add.70210)
Supplement: Supplementary file 1 — Figure SA1 Example of neutral adverts. Figure SA2 Example page from the experimental online supermarket. Table SA1 Number of products available by department and aisle in the experimental online supermarket. Table SC1 Logistic regression models for the selection of alcoholic and NoLo version of the advertised product by group. Table SC2 Generalised linear mixed‐effect models (GLMMs) for the selection of alcoholic and NoLo version of the advertised product by group with brands as random effect. Table SC3 Logistic regression models for the selection of any alcoholic and NoLo products regardless of brands by group. Table SC4 Paired‐sample t‐tests for the differences in support for each policy scenarios. Table SC5 Linear regression models for energy (kcal) selected in basket. Table SC6 Number (proportion) of participants in NLG by risk of alcohol dependence categories. Table SC7 Logistic regression models with an interaction term between cohort (NLG vs UG) and risk of alcohol dependence categories. Figure SC1 Selection of any alcoholic or NoLo products regardless of brands in NLG by risk of alcohol dependence measured by AUDIT‐C. NLG: NoLo group. UG: unrelated advert group. [file ADD-121-388-s001.docx]

Supplementary Appendices

Contents

[Supplementary Appendix A 2](#_Toc210829872)

[Figure A1 Example of neutral adverts 2](#_Toc210829873)

[Figure A2 Example page from the experimental online supermarket 2](#_Toc210829874)

[Table A1 Number of products available by department and aisle in the experimental online supermarket 3](#_Toc210829875)

[Supplementary Appendix B 5](#_Toc210829876)

[Sample size calculation 5](#_Toc210829877)

[Measures for demographic characteristics and lifestyle factors 5](#_Toc210829878)

[Table B1 Measures and interpretation of response for recall of advertisement, brands, and products 7](#_Toc210829879)

[Supplementary Appendix C 9](#_Toc210829880)

[Table C1 Logistic regression models for the selection of alcoholic and NoLo version of the advertised product by group 9](#_Toc210829881)

[Table C2 Generalised linear mixed-effect models (GLMMs) for the selection of alcoholic and NoLo version of the advertised product by group with brands as random effect 9](#_Toc210829882)

[Table C3 Logistic regression models for the selection of any alcoholic and NoLo products regardless of brands by group 9](#_Toc210829883)

[Table C4 Paired-sample t-tests for the differences in support for each policy scenarios 9](#_Toc210829884)

[Table C5 Linear regression models for energy (Kcal) selected in basket 10](#_Toc210829885)

[Table C6 Number (proportion) of participants in NLG by risk of alcohol dependence categories 10](#_Toc210829886)

[Table C7 Logistic regression models with an interaction term between cohort (NLG vs UG) and risk of alcohol dependence categories 11](#_Toc210829887)

[Figure C1 Selection of any alcoholic or NoLo products regardless of brands in NLG by risk of alcohol dependence measured by AUDIT-C. NLG: NoLo group. UG: unrelated advert group. Covariates were set to be at mean (age) or reference levels (female, White British, no university degrees, and household income of £15K-£24K). 12](#_Toc210829888)

# Supplementary Appendix A


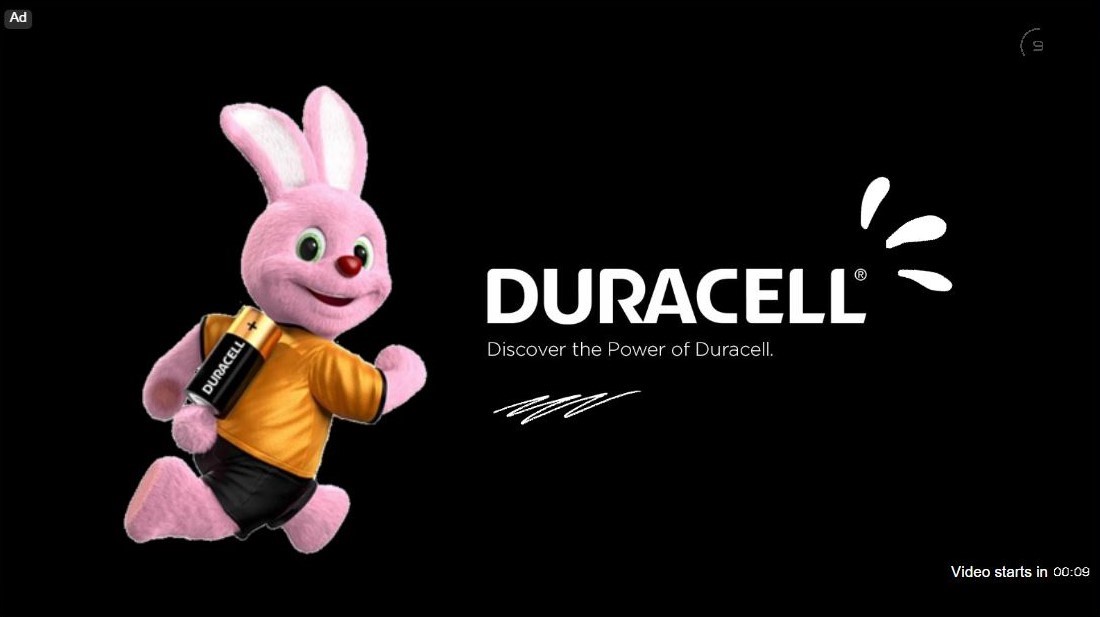


## Figure A1 Example of neutral adverts


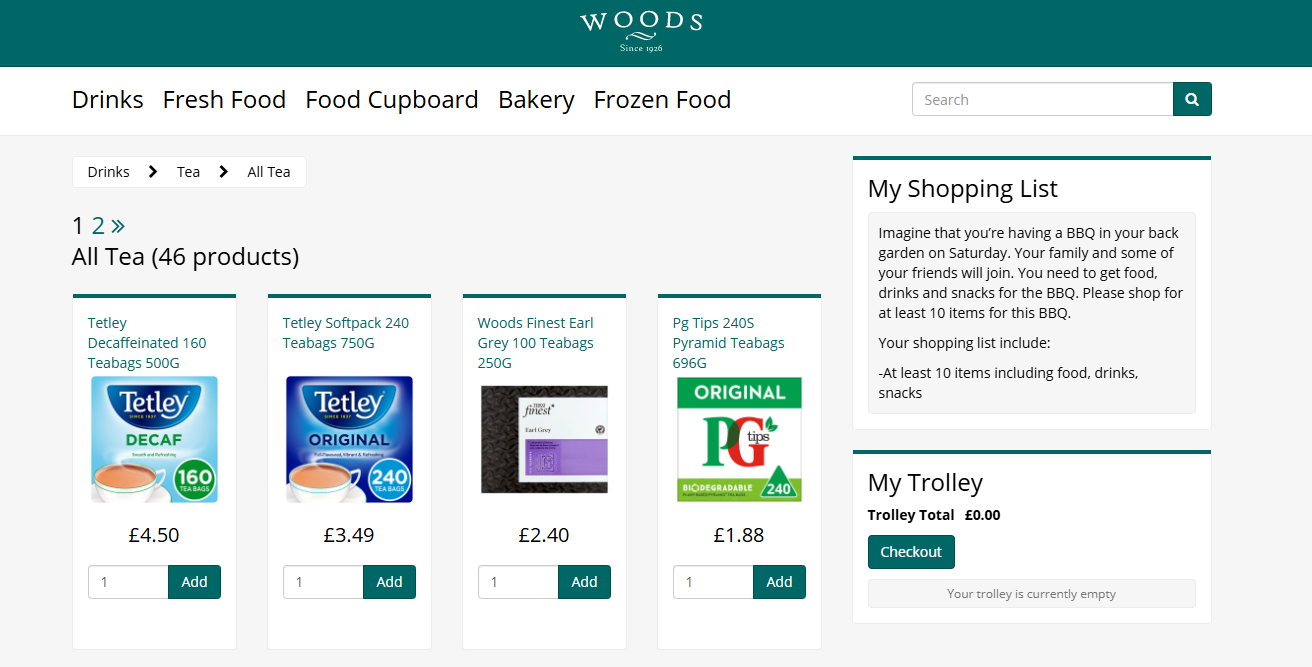


## Figure A2 Example page from the experimental online supermarket

Note. Each page presents a maximum of 28 products in total.

## Table A1 Number of products available by department and aisle in the experimental online supermarket

| **Department** | **Aisle** | **Number of products** |
| --- | --- | --- |
| Bakery | Bakery Free From | 69 |
| Bakery | Bread & Rolls | 105 |
| Bakery | Cakes, Cake Bars, Slices & Pies | 217 |
| Bakery | Croissants, Brioche & Pastries | 29 |
| Bakery | Crumpets, Muffins & Pancakes | 13 |
| Bakery | Doughnuts, Cookies & Muffins | 28 |
| Bakery | From Our Bakery | 4 |
| Bakery | Teacakes, Fruit Loaves & Scones | 2 |
| Bakery | Wraps, Pittas, Naan & Thins | 8 |
| Drinks | Adult Soft Drinks & Mixers | 62 |
| Drinks | Alcohol Free & Low Alcohol Drinks | 14 |
| Drinks | Alcohol Gift Sets | 1 |
| Drinks | Beer & Cider | 194 |
| Drinks | Coffee | 61 |
| Drinks | Fizzy & Soft Drinks | 66 |
| Drinks | Hot Chocolate & Malted Drinks | 34 |
| Drinks | Juices & Smoothies | 103 |
| Drinks | Kids Drinks | 34 |
| Drinks | Milkshake | 36 |
| Drinks | On The Go Drinks | 44 |
| Drinks | Spirits & Liqueurs | 60 |
| Drinks | Sports, Energy & Wellness Drinks | 23 |
| Drinks | Squash & Cordial | 60 |
| Drinks | Tea | 46 |
| Drinks | Water | 23 |
| Drinks | Wine | 28 |
| Food Cupboard | Biscuits & Cereal Bars | 242 |
| Food Cupboard | Cereals | 229 |
| Food Cupboard | Chocolate, Sweets, Mints & Chewing Gum | 452 |
| Food Cupboard | Cooking Ingredients | 326 |
| Food Cupboard | Cooking Sauces, Meal Kits & Sides | 386 |
| Food Cupboard | Crackers & Crispbreads | 86 |
| Food Cupboard | Crisps, Snacks, Nuts & Popcorn | 244 |
| Food Cupboard | Desserts | 130 |
| Food Cupboard | Dried Fruit, Nuts, Nutrient Powders & Seeds | 95 |
| Food Cupboard | Dried Pasta, Rice, Noodles & Cous Cous | 130 |
| Food Cupboard | Free From Range | 120 |
| Food Cupboard | Home Baking | 191 |
| Food Cupboard | Jams, Sweet & Savoury Spreads | 110 |
| Food Cupboard | Table Sauces, Olives, Pickles & Chutneys | 296 |
| Food Cupboard | Tinned & Packaged Ready Meals | 190 |
| Food Cupboard | Tins, Cans & Packets | 425 |
| Food Cupboard | World Foods | 353 |
| Fresh Food | Cheese | 188 |
| Fresh Food | Chilled Desserts | 194 |
| Fresh Food | Chilled Fish, Seafood & Alternatives | 79 |
| Fresh Food | Chilled Soup, Sandwiches & Salad Pots | 245 |
| Fresh Food | Cooked Meats, Antipasti & Dips | 211 |
| Fresh Food | Counters | 7 |
| Fresh Food | Fresh Fruit | 142 |
| Fresh Food | Fresh Meat, Poultry & Alternatives | 341 |
| Fresh Food | Fresh Pizza, Pasta & Garlic Bread | 61 |
| Fresh Food | Fresh Salad & Sandwich Fillers | 89 |
| Fresh Food | Fresh Vegetables | 141 |
| Fresh Food | Juice & Smoothies | 72 |
| Fresh Food | Milk, Butter & Eggs | 183 |
| Fresh Food | Pies, Pasties, Quiches & Snacking | 164 |
| Fresh Food | Ready Meals | 248 |
| Fresh Food | World Foods | 21 |
| Fresh Food | Yoghurts | 253 |
| Frozen Food | Desserts | 41 |
| Frozen Food | Frozen Breakfast, Fruit & Pastry | 19 |
| Frozen Food | Frozen Chips, Onion Rings, Potatoes & Rice | 52 |
| Frozen Food | Frozen Fish, Seafood & Alternatives | 81 |
| Frozen Food | Frozen Free From | 2 |
| Frozen Food | Frozen Meat, Poultry & Alternatives | 167 |
| Frozen Food | Frozen Pies | 28 |
| Frozen Food | Frozen Pizza & Garlic Bread | 50 |
| Frozen Food | Frozen Ready Meals | 105 |
| Frozen Food | Frozen Sausage Rolls, Pasties & Snacks | 27 |
| Frozen Food | Frozen Vegetables & Herbs | 12 |
| Frozen Food | Frozen World Foods & Halal | 6 |
| Frozen Food | Frozen Yorkshire Puddings & Stuffing | 7 |
| Frozen Food | Ice Cream & Lollies | 240 |

# Supplementary Appendix B

## Sample size calculation

To aid sample size estimation, we conducted a pilot trial, where participants (n=200) followed the same study procedure as the main study. In the pilot trial, we found that the proportion of people in the alcohol group who selected advertised product was 0.04, the proportion of people in the alcohol-free group who selected the advertised product was 0.05. Based on this, we powered the study for an effect size for a logistic regression predicting the selection of advertised product by group of f^2^=0.026. Using R, we calculated the required sample size to achieve 90% power, 5% alpha, would be 406 participants per intervention group, and 598 in the neutral advert group (assuming an 80% power due to the increased number of comparisons against the neutral advert group). Allowing for 15% attrition, a total sample size of 1640 would be needed (467 in each product advert group and 688 in the neutral advert group) for this study.

## Measures for demographic characteristics and lifestyle factors

1. What is your sex? A question about gender identity will follow.

| Male |
| --- |
| Female |
| Prefer not to say |

1. Which of the following best describes your gender?

| Man |
| --- |
| Woman |
| Non-binary |
| Prefer to self-describe (specify, if you wish) |
| Prefer not to say |

1. Please tell us your age (in years)

[FREE TEXT ANSWER]

1. How would you describe your ethnicity?

| Bangladeshi |
| --- |
| Chinese |
| Indian |
| Pakistani |
| Any other Asian background (specify, if you wish) |
| African |
| Caribbean |
| Any other black, African or Caribbean background (specify, if you wish) |
| African |
| White and black Caribbean |
| White and Asian |
| White and black African |
| Any other mixed or multiple ethnic background (specify, if you wish) |
| English, Welsh, Scottish, Northern Irish or British |
| Irish |
| Gypsy or Irish Traveller |
| Roma |
| Any other white background (specify, if you wish) |
| Arab |
| Any other ethnic group (specify, if you wish) |
| Prefer not to say |

1. Can you tell us the **highest** educational or school qualification you have obtained?

| No qualifications |
| --- |
| Up to 4 or less GCSEs  *(Level 1 and entry level qualifications: 1 to 4 GCSEs grade A* to C , Any GCSEs at other grades, O levels or CSEs (any grades), 1 AS level, NVQ level 1, Foundation GNVQ, Basic or Essential Skills) |
| Up to 5GCSEs, or 1 A Level or Apprenticeships  *(Level 2 qualifications: 5 or more GCSEs (A* to C or 9 to 4), O levels (passes), CSEs (grade 1), School Certification, 1 A level, 2 to 3 AS levels, VCEs, Intermediate or Higher Diploma, Welsh Baccalaureate Intermediate Diploma, NVQ level 2, Intermediate GNVQ, City and Guilds Craft, BTEC First or General Diploma, RSA Diploma; or Apprenticeship; or vocational or work-related qualifications, other qualifications achieved in England or Wales, qualifications achieved outside England or Wales (equivalent not stated or unknown)) |
| Up to 2+ A levels  *(Level 3 qualifications: 2 or more A levels or VCEs, 4 or more AS levels, Higher School Certificate, Progression or Advanced Diploma, Welsh Baccalaureate Advance Diploma, NVQ level 3; Advanced GNVQ, City and Guilds Advanced Craft, ONC, OND, BTEC National, RSA Advanced Diploma) |
| Up to University Degree  *(Level 4 qualifications or above: degree (BA, BSc), higher degree (MA, PhD, PGCE), NVQ level 4 to 5, HNC, HND, RSA Higher Diploma, BTEC Higher level, professional qualifications (for example, teaching, nursing, accountancy)) |
| Prefer not to say |

1. What’s your household income per year (before tax)?

| Less than £15K |
| --- |
| Between £15K and £24K |
| Between £24K and £32K |
| Between £32K and £43K |
| Between £43K and £66K |
| £66K or above |
| Prefer not to say |

1. How many people live at your house, including you?

| Please specify (in numbers) [FREE TEXT ANSWER] |
| --- |
| Prefer not to say |

1. On average, how much (in £) do you spend on supermarket shopping per week? (Please write down a number)

| Please specify (in numbers) [FREE TEXT ANSWER] |
| --- |
| Prefer not to say |

1. How often, on average over the past year, have you shopped online for food or groceries to be delivered to you (e.g. Tesco.com, Ocado.com, mysupermarket.co.uk)?

| Never or not in the last year |
| --- |
| 1-3 times in the last year |
| 4-11 times in the last year |
| 1-3 per month |
| Once per week or more often |
| Prefer not to say |

1. Have you had any alcoholic drinks (e.g., beer, wine, cocktail) in the past month?

| Yes |
| --- |
| No |
| Prefer not to say |

10a. (If answered Yes to Question 10) Have you purchased any alcoholic drinks (e.g., a pint of beer at a pub) in the past month?

| Yes |
| --- |
| No |
| Prefer not to say |

## Table B1 Measures and interpretation of response for recall of advertisement, brands, and products

| Variables | Survey question | Response | Interpretation of response |
| --- | --- | --- | --- |
| Recall of advertisement | While you were watching the video earlier, did you notice any advertisement? | Yes | Recall advertisement |
|  |  | No | Did not recall advertisement |
|  |  | I’m not sure | Did not recall advertisement |
| Recall of brand and product | (if selected “Yes” for the previous question) Which of the following brands was the advertisement about? | Gordon’s Gin | If participants selected the correct product that was advertised to them, they were recorded as being able to recall both the brand and product.  If participants selected the correct brand, but not product that was advertised to them, they were recorded as being able to recall the brand but not product.  If participants selected the wrong brand that was advertised to them, they were recorded as not being able to recall the brand or product. |
|  |  | Alcohol-free 0.0% Gordon’s Gin |  |
|  |  | Guinness Draught |  |
|  |  | Alcohol-free 0.0% Guinness |  |
|  |  | Heineken Lager Beer |  |
|  |  | Alcohol-free 0.0% Heineken |  |
|  |  | Carlsberg Lager Beer |  |
|  |  | Alcohol-free 0.0% Carlsberg |  |
|  |  | Duracell battery |  |

# Supplementary Appendix C

## Table C1 Logistic regression models for the selection of alcoholic and NoLo version of the advertised product by group

| Outcome | Group | Reference | odds ratio | 95%CI | Unadjusted *p* value | BH-adjusted *p* value |
| --- | --- | --- | --- | --- | --- | --- |
| Alcoholic version | AG | UG | 1.62 | (1.02 to 2.52) | 0.035 | 0.11 |
|  | NLG | UG | 1.48 | (0.94 to 2.33) | 0.089 | 0.13 |
|  | NLG | AG | 0.92 | (0.58 to 1.44) | 0.71 | 0.71 |
| NoLo version | AG | UG | 1.43 | (0.76 to 2.68) | 0.26 | 0.26 |
|  | NLG | UG | 2.18 | (1.24 to 3.91) | 0.0072 | 0.022 |
|  | NLG | AG | 1.52 | (0.86 to 2.76) | 0.16 | 0.24 |

Note. AG: alcohol group, NLG: NoLo group, UG: control.

## Table C2 Generalised linear mixed-effect models (GLMMs) for the selection of alcoholic and NoLo version of the advertised product by group with brands as random effect

| Outcome | Group | Reference | odds ratio | 95%CI | Unadjusted *p* value | BH-adjusted *p* value |
| --- | --- | --- | --- | --- | --- | --- |
| Alcoholic version | AG | UG | 1.63 | (1.04 to 2.54) | 0.033 | 0.099 |
|  | NLG | UG | 1.45 | (0.92 to 2.28) | 0.11 | 0.17 |
|  | NLG | AG | 0.89 | (0.56 to 1.40) | 0.62 | 0.62 |
| NoLo version | AG | UG | 1.43 | (0.77 to 2.66) | 0.26 | 0.26 |
|  | NLG | UG | 2.13 | (1.21 to 3.76) | 0.0093 | 0.028 |
|  | NLG | AG | 1.49 | (0.84 to 2.66) | 0.18 | 0.26 |

Note. AG: alcohol group, NLG: NoLo group, UG: control.

## Table C3 Logistic regression models for the selection of any alcoholic and NoLo products regardless of brands by group

| Outcome | Group | Reference | odds ratio | 95%CI | Unadjusted *p* value | BH-adjusted *p* value |
| --- | --- | --- | --- | --- | --- | --- |
| Any alcoholic product | AG | UG | 1.23 | (0.94, 1.61) | 0.13 | 0.40 |
|  | NLG | UG | 1.08 | (0.83, 1.41) | 0.56 | 0.56 |
|  | NLG | AG | 0.88 | (0.66, 1.18) | 0.40 | 0.56 |
| Any NoLo product | AG | UG | 1.16 | (0.87, 1.53) | 0.32 | 0.32 |
|  | NLG | UG | 1.41 | (1.07, 1.85) | 0.015 | 0.045 |
|  | NLG | AG | 1.22 | (0.91, 1.64) | 0.19 | 0.29 |

Note. AG: alcohol group, NLG: NoLo group, UG: control.

## Table C4 Paired-sample t-tests for the differences in support for each policy scenarios

| Policy scenario 1 | Policy scenario 2 | t | df | Bonferroni adjusted *p* |
| --- | --- | --- | --- | --- |
| Policy support^1^: *alcohol advertising allowed* | Policy support^1^: *brand advertising allowed* | -9.72 | 1237 | < 0.001 |
| Policy support^1^: *alcohol advertising allowed* | Policy support^1^: *NoLo advertising to young* | 44.08 | 1628 | < 0.001 |
| Policy support^1^: *alcohol advertising allowed* | Policy support^1^: *restrict alcohol advertising* | 17.66 | 1627 | < 0.001 |
| Policy support^1^: *alcohol advertising allowed* | Policy support^1^: *restrict brand advertising* | 20.52 | 1623 | < 0.001 |
| Policy support^1^: *brand advertising allowed* | Policy support^1^: *NoLo advertising to young* | 44.29 | 1237 | < 0.001 |
| Policy support^1^: *brand advertising allowed* | Policy support^1^: *restrict alcohol advertising* | 25.388 | 1237 | < 0.001 |
| Policy support^1^: *brand advertising allowed* | Policy support^1^: *restrict brand advertising* | 27.61 | 1234 | < 0.001 |
| Policy support^1^: *NoLo advertising to young* | Policy support^1^: *restrict alcohol advertising* | -19.17 | 1627 | < 0.001 |
| Policy support^1^: *NoLo advertising to young* | Policy support^1^: *restrict brand advertising* | -16.23 | 1623 | < 0.001 |
| Policy support^1^: *restrict alcohol advertising* | Policy support^1^: *restrict brand advertising* | 5.28 | 1623 | < 0.001 |

^1^ Sums of responses to two questions (“Do you support or oppose this policy?” and “How acceptable do you find this policy?”), on 7-pt scales, with higher scores representing higher support.

## Table C5 Linear regression models for energy (Kcal) selected in basket

| Outcome | Group | Reference | **coefficient (β)** | 95%CI | Unadjusted *p* value | BH-adjusted *p* value |
| --- | --- | --- | --- | --- | --- | --- |
| Energy (Kcal) from alcoholic version of advertised products | AG | UG | 0.18 | (-0.018, 0.38) | 0.075 | 0.15 |
|  | NLG | UG | 0.17 | (-0.031, 0.37) | 0.097 | 0.15 |
|  | NLG | AG | -0.013 | (-0.23, 0.21) | 0.91 | 0.91 |
| Energy (Kcal) from NoLo version of advertised products | AG | UG | 0.068 | (-0.077, 0.21) | 0.36 | 0.36 |
|  | NLG | UG | 0.20 | (0.055, 0.34) | 0.0070 | 0.021 |
|  | NLG | AG | 0.13 | (-0.027, 0.29) | 0.10 | 0.15 |
| Total energy (Kcal) | AG | UG | 0.038 | (-0.024 to 0.10) | 0.23 | 0.15 |
|  | NLG | UG | 0.040 | (-0.022 to 0.10) | 0.20 | 0.15 |
|  | NLG | AG | 0.0021 | (-0.065 to 0.069) | 0.95 | 0.91 |

Note. AG: alcohol group, NLG: NoLo group, UG: control.

## Table C6 Number (proportion) of participants in NLG by risk of alcohol dependence categories

| Categories | Number (%) in NLG | Number (%) in UG |
| --- | --- | --- |
| Low risk | 188 (40%) | 273 (40%) |
| Increasing risk | 165 (35%) | 249 (36%) |
| Higher risk | 84 (18%) | 142 (20%) |
| Possible dependence | 31 (7%) | 28 (4%) |

Note. NLG: NoLo group. UG: unrelated advert group

## Table C7 Logistic regression models with an interaction term between cohort (NLG vs UG) and risk of alcohol dependence categories

| Outcome | Terms | odds ratio | 95%CI | *p* value | BH-adjusted *p* value |
| --- | --- | --- | --- | --- | --- |
| Any alcoholic product | Cohort: NLG | 1.01 | (0.66, 1.54) | 0.98 | 0.98 |
|  | AUDIT-C: Increasing risk | 1.44 | (0.96, 2.18) | 0.08 | 0.38 |
|  | AUDIT-C: Higher risk | 1.44 | (0.89, 2.37) | 0.14 | 0.54 |
|  | AUDIT-C: Possible Dependence | 1.41 | (0.59, 3.77) | 0.47 | 0.72 |
|  | Age | 1.02 | (1.01, 1.03) | 0.00 | 0.04 |
|  | Gender: men | 0.83 | (0.62, 1.11) | 0.20 | 0.54 |
|  | Ethnicity: Asian or Asian British | 0.49 | (0.27, 0.89) | 0.02 | 0.19 |
|  | Ethnicity: Black, black British, Caribbean or African | 0.59 | (0.35, 1.01) | 0.05 | 0.34 |
|  | Ethnicity: Mixed or multiple ethnic groups | 0.71 | (0.39, 1.32) | 0.27 | 0.59 |
|  | Ethnicity: Other | 1.04 | (0.10, 22.84) | 0.97 | 0.98 |
|  | Education: University degrees | 0.80 | (0.57, 1.14) | 0.21 | 0.54 |
|  | Household income: < £15K | 0.83 | (0.45, 1.54) | 0.54 | 0.72 |
|  | Household income: £24K-£32K | 0.91 | (0.54, 1.51) | 0.71 | 0.84 |
|  | Household income: £32K-£43K | 1.28 | (0.76, 2.16) | 0.35 | 0.63 |
|  | Household income: £43K-£66K | 1.42 | (0.86, 2.34) | 0.17 | 0.54 |
|  | Household income: >=£66K | 1.23 | (0.74, 2.02) | 0.42 | 0.71 |
|  | NLG*Increasing risk | 0.89 | (0.47, 1.67) | 0.71 | 0.84 |
|  | NLG*Higher risk | 1.52 | (0.67, 3.59) | 0.33 | 0.63 |
|  | NLG*Possible dependence | 1.56 | (0.40, 6.49) | 0.53 | 0.72 |
| Any NoLo product | Cohort: NLG | 1.18 | (0.75, 1.86) | 0.47 | 0.83 |
|  | AUDIT-C: Increasing risk | 0.86 | (0.55, 1.33) | 0.49 | 0.83 |
|  | AUDIT-C: Higher risk | 0.92 | (0.54, 1.55) | 0.76 | 0.83 |
|  | AUDIT-C: Possible Dependence | 0.47 | (0.11, 1.42) | 0.23 | 0.77 |
|  | Age | 1.01 | (1.00, 1.02) | 0.12 | 0.53 |
|  | Gender: men | 0.91 | (0.67, 1.23) | 0.52 | 0.83 |
|  | Ethnicity: Asian or Asian British | 0.72 | (0.34, 1.43) | 0.37 | 0.83 |
|  | Ethnicity: Black, black British, Caribbean or African | 2.02 | (1.15, 3.50) | 0.01 | 0.09 |
|  | Ethnicity: Mixed or multiple ethnic groups | 0.91 | (0.44, 1.78) | 0.79 | 0.83 |
|  | Ethnicity: Other | 1.60 | (0.07, 17.32) | 0.71 | 0.83 |
|  | Education: University degrees | 1.81 | (1.25, 2.62) | 0.00 | 0.02 |
|  | Household income: < £15K | 0.90 | (0.45, 1.75) | 0.77 | 0.83 |
|  | Household income: £24K-£32K | 0.82 | (0.48, 1.41) | 0.47 | 0.83 |
|  | Household income: £32K-£43K | 1.01 | (0.60, 1.72) | 0.97 | 0.97 |
|  | Household income: £43K-£66K | 0.93 | (0.56, 1.54) | 0.76 | 0.83 |
|  | Household income: >=£66K | 0.77 | (0.46, 1.31) | 0.33 | 0.83 |
|  | NLG*Increasing risk | 1.65 | (0.86, 3.19) | 0.13 | 0.53 |
|  | NLG*Higher risk | 1.21 | (0.54, 2.70) | 0.65 | 0.83 |
|  | NLG*Possible dependence | 1.60 | (0.35, 8.87) | 0.56 | 0.83 |

Note. NLG: NoLo group. UG: unrelated advert group


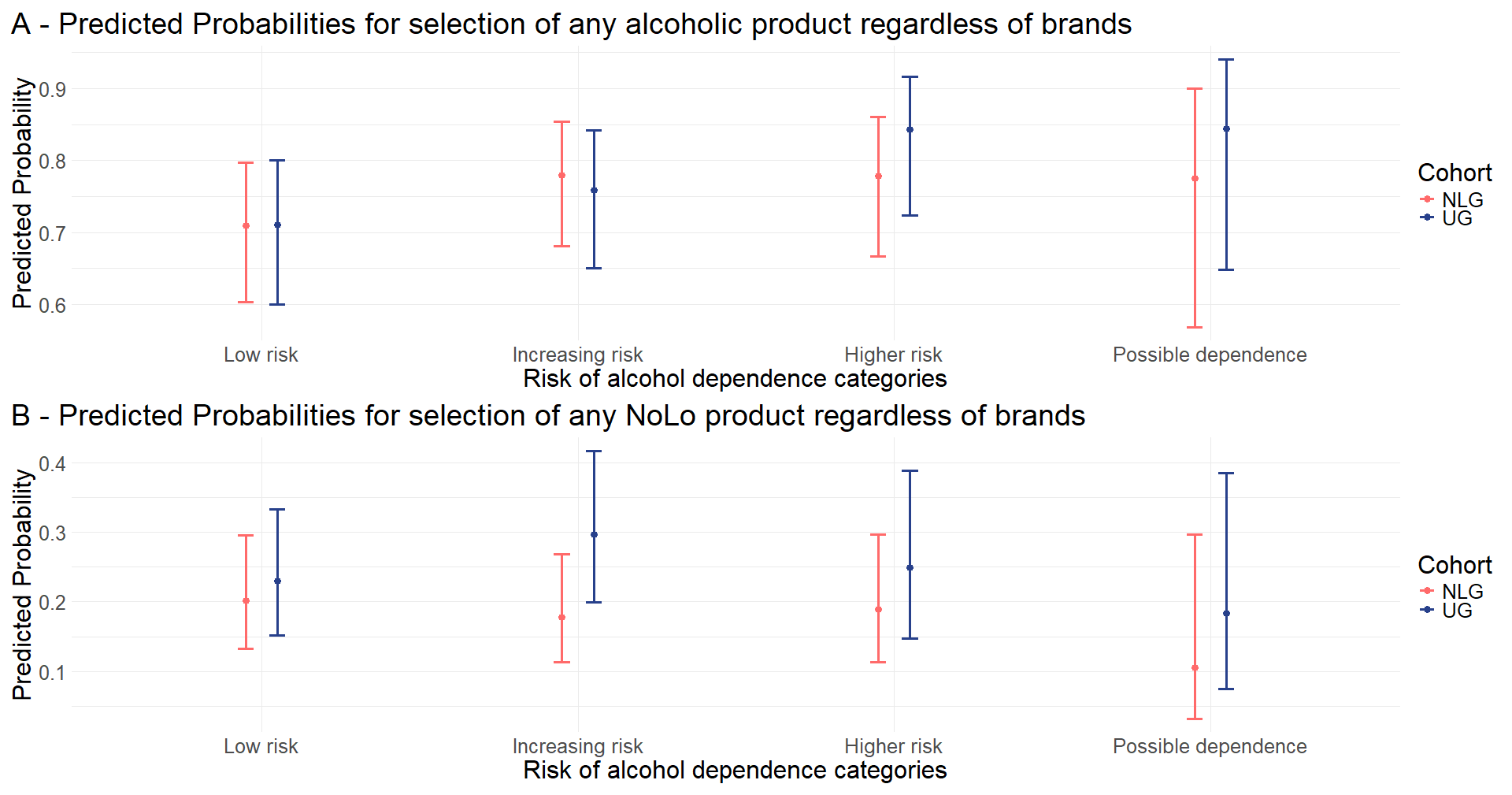


## Figure C1 Selection of any alcoholic or NoLo products regardless of brands in NLG by risk of alcohol dependence measured by AUDIT-C. NLG: NoLo group. UG: unrelated advert group. Covariates were set to be at mean (age) or reference levels (female, White British, no university degrees, and household income of £15K-£24K).
